# Comparison of Phenotypic and Genotypic Characterization Methods for the Detection of Methicillin-Resistant Staphylococcus Aureus

**DOI:** 10.7759/cureus.23396

**Published:** 2022-03-22

**Authors:** Rajeswarie S, Pradha V, Annet Olinda D'Souza, R Vinod

**Affiliations:** 1 Microbiology, Al-Azhar Medical College & Super Specialty Hospital, Thodupuzha, IND; 2 Microbiology, Sri Lakshmi Narayana Institute of Medical Sciences, Puducherry, IND; 3 Microbiology, Udupi District Hospital, Udupi, IND; 4 Microbiology, Sri Venkateshwaraa Medical College Hospital and Research Centre, Puducherry, IND

**Keywords:** d test, mannitol salt agar, polymerase chain reaction, cefoxitin disk diffusion, methicillin-resistant staphylococcus aureus

## Abstract

Introduction

Methicillin-resistant Staphylococcus aureus (MRSA) is associated with high morbidity and mortality due to the development of antimicrobial resistance secondary to irrational use of antibiotics, nonadherence to infection control practices, and increased use of intravascular devices in healthcare systems. Detection of MRSA is critical in clinical microbiology laboratories as it helps identify MRSA carriers and avoid treatment failure in patients. Hence, this study compared various phenotypic methods with the standard genotyping method to determine a method that permits rapid and accurate detection of MRSA.

Materials & Methods

*Staphylococcus aureus* (*S. aureus*) was initially identified based on colony morphology, Gram staining, standard biochemical tests, and antibiotic susceptibility using disk diffusion. MRSA was identified based on the detection of the mecA gene by polymerase chain reaction (PCR) and subsequent gel electrophoresis. Disk diffusion using cefoxitin or oxacillin and mannitol salt agar with 6-µg/ml oxacillin were used for phenotypic detection of MRSA. The D test was used to detect inducible clindamycin resistance in *S. aureus* isolates.

Results

Of the 100 *S. aureus* isolates analyzed, 37% were identified as MRSA by PCR and the cefoxitin disk diffusion method; however, only 31% were detected by the oxacillin disk diffusion method and 29% by the mannitol salt agar method. The sensitivity of the cefoxitin disk diffusion test, oxacillin disk diffusion, and mannitol salt agar methods was 86.05%, 83.78%, and 70.73%, respectively. Specificity was 100% for all the three phenotypic methods (p < 0.001). Notably, inducible clindamycin resistance was found in 37.2% of the MRSA isolates, indicating potential challenges in treatment.

Conclusion

Among the three phenotypic methods tested, the cefoxitin disk diffusion method had 100% sensitivity and specificity, which is similar to that of PCR-based MRSA detection. Hence, the cefoxitin disk diffusion method is recommended for use in clinical laboratories, where molecular methods are not available as it is both cost-effective and easy to perform.

## Introduction

*Staphylococcus aureus *(*S. aureus*) is a widespread organism that can be isolated from various clinical specimens and causes a wide spectrum of diseases like furunculosis, cellulitis, abscess, pyoderma, toxic shock syndrome, staphylococcal scalded skin syndrome, endocarditis, septicemia, and pneumonia [1]. Moreover, asymptomatic *S. aureus* colonizers can transmit the bacteria to individuals in both healthcare and community settings, which is a significant contributor to a prolonged hospital stay, poor clinical outcomes, and greater healthcare costs among surgical patients [2].

Beta-lactam antibiotics, such as penicillin, disrupt bacterial cell wall synthesis and inhibit growth by binding to the enzymatic site of penicillin-binding proteins (PBPs) [3]. Penicillin was the first drug against which *S. aureus* developed resistance; this was mediated by a beta-lactamase enzyme called penicillinase, which rendered penicillin ineffective [3]. Hence, in 1959, a penicillinase-resistant antibiotic, methicillin, was introduced. However, in 1961, hospitals reported the emergence of a new resistant strain called methicillin-resistant Staphylococcus aureus (MRSA), which was later reported from the community as well [3]. Mechanistically, methicillin resistance is due to the acquisition of the mecA gene, which is present in the mobile small cassette chromosome and is responsible for the change from PBP to PBP2a in the cell wall of MRSA.

Given the resistance patterns of MRSA, macrolide-lincosamide-streptogramin B class of antibiotics is used to treat MRSA infections. Clindamycin, an important drug, is useful for treating pneumonia and soft-tissue and musculoskeletal infections caused by MRSA, but the development of resistance to clindamycin during therapy has discouraged some clinicians from prescribing it. Current strategies employed to control MRSA infection include simple and rapid detection of methicillin-resistant cases, identification of carriers of MRSA, investigation of factors responsible for colonization, and prompt treatment measures.

Multiple phenotypic tests are commonly performed in clinical laboratories for detecting MRSA, such as cefoxitin disk diffusion, oxacillin disk diffusion, and mannitol salt agar screen, but the gold standard is the genotypic detection of the mecA gene. As genotypic methods may not always be available, this study compared MRSA detection by three commonly available phenotypic methods with the genotypic method.

## Materials and methods

This prospective cohort study was conducted for a period of one year, from October 2013 to October 2014, in the Department of Microbiology at our institute. This study included a total of 100 isolates of *S. aureus*, which were obtained from different clinical samples and had been identified by culture and biochemical tests [4].

Ethical clearance

Approval from Institutional Research Board and Institutional Ethical Committee (IEC Ref ID: SVMCH/IEC/2013/31) was obtained before study commencement.

Phenotypic methods of MRSA detection

Cefoxitin Disk Diffusion Test

A 0.5 McFarland standard suspension of the isolate was lawn cultured on a Mueller-Hinton agar plate (Hi-Media, India) with a 30-µg cefoxitin disk. All plates were incubated at 37°C overnight. A zone size of 22 mm or more was defined as sensitive, and zone sizes less than 22 mm were considered MRSA. *Staphylococcus aureus* ATCC-25923 and MRSA-43300 were used as control cultures [5].

Oxacillin Disk Diffusion Test

A 0.5 McFarland standard suspension of the isolate was lawn cultured on an MHA plate (Hi-Media, India) with a 1-µg oxacillin disk. All plates were incubated at 37°C overnight. A zone size of 13 mm or more was defined as sensitive, 11-12 mm as intermediate, and <10 mm as MRSA. *Staphylococcus aureus* ATCC-25923 and MRSA-43300 were used as control cultures [5].

Mannitol Salt Agar With 6-µg Oxacillin

A bacterial inoculum of each strain was adjusted to 0.5 McFarland, and one drop of each strain was inoculated on mannitol salt agar containing 6-µg/ml of oxacillin. Plates were incubated at 35°C for 24 h. The presence of yellow colonies indicated MRSA, whereas no growth was defined as methicillin-sensitive Staphylococcus aureus (MSSA). ATCC-25923 and MRSA-43300 were used as control cultures [6].

Polymerase Chain Reaction Detection of MRSA by mecA

All suspected MRSA isolates were confirmed by polymerase chain reaction (PCR) and subsequent agarose gel electrophoresis, which is the gold standard test [7]. A DNA extraction kit containing a spin column and an amplification kit for PCR and agarose gel electrophoresis was purchased from HELINI Biomolecules, Chennai. The amplification kit consisted of forward and reverse primers for mecA, 5ʹ GTT GAA ATG ACT GAA CGT CCCG ATA A 3ʹ and 5ʹ CCA ATT CCA CAT TGT TTC GGT CTA A 3ʹ, which would yield an amplification product of 310 kbp [7]. Each PCR tube contained a 15-µl master mix and 5-µl extracted DNA. PCR conditions were initial annealing at 95°C for 5 min, denaturation at 95°C for 45 s, annealing at 54°C for 45 s, extension at 72°C for 30 s for 35 cycles, and final extension at 72°C for 3 min. Next, 20 µl of each sample, 15 µl of negative control, and 15 µl of positive control were loaded onto a 1% agarose gel containing ethidium bromide. A 100-bp molecular weight ladder was used to identify the amplified product.

“D-Test" for Detecting Inducible Clindamycin Resistance

A 0.5 McFarland suspension of *S. aureus* isolates was inoculated onto a Muller-Hinton agar plate. Clindamycin (2 µg) and erythromycin (15 µg) disks were placed at an edge-to-edge distance of 15-20 mm and incubated overnight at 37°C. *S. aureus* isolates showing resistance to erythromycin (zone size ≤ 13 mm) and a clear, D-shaped zone of inhibition around the clindamycin disk were defined as having inducible clindamycin resistance, i.e., the D phenotype [8].

Statistical analysis

A parametric test was used for analysis as the data followed a normal distribution. Comparison of test of significance was done by the analysis of variance (ANOVA). Scheffe’s test was used for post hoc analysis. The level of significance was taken as 5%. SPSS Inc. Released 2007. SPSS for Windows, Version 16.0. Chicago, SPSS Inc. was used. 

## Results

In 100 isolates of *S. aureus*, 37% of MRSA and 63% MSSA were detected by PCR. Out of 100 *S. aureus* isolates by phenotypic methods, 37% of MRSA was detected by the Cefoxitin disc diffusion method, 31% of MRSA by oxacillin disc diffusion method, and 29% of MRSA by Mannitol salt agar with Oxacillin. The cefoxitin disc diffusion test, Oxacillin disc diffusion, Mannitol salt agar with 6μg oxacillin have a sensitivity of 86.05%, 83.78%, and 70.73%, respectively, with 100% specificity for all the three phenotypic methods with p-value <0.001, which is significant (Table 1).

**Table 1:** Comparison of phenotypic and genotypic method in detection of MRSA

Sl. No	Method	MRSA	Sensitivity	Specificity
1.	Cefoxitin Disc Diffusion	37	100%	100%
2.	Oxacillin Disc Diffusion	31	83.78%	100%
3.	Mannitol Salt Agar With 6μg Oxacillin	29	70.73%	100%
4.	Polymerase Chain Reaction	37	100%	100%

In this study, out of 100 *S. aureus*, 24 iMLSB was detected, among which 14 (37.8%) were MRSA isolates and 10 (15.8%) MSSA isolates (Table 2).

**Table 2:** Inducible clindamycin resistance in MSSA and MRSA by D-test i-MLSB: inducible macrolide-lincosamide-streptogramin B

i-MLSB*	MRSA	MSSA
PRESENT	14 (37.8%)	10 (15.8%)
ABSENT	23 (62.1%)	53 (84.1%)
TOTAL	37	63

## Discussion

The recent increase in methicillin-resistant Staphylococcus aureus has posed a great threat to the clinician in treating the infections caused by *S. aureus*. The appropriate identification of MRSA from that of MSSA is of prime importance since the prognosis and treatment options differ from each other.

In this study, the rate of methicillin-resistant Staphylococcus aureus was found to be 37%. This is in accordance with the various studies in India, which range from 25% to 50% [9].

We compared the three commonly available phenotypic methods and analyzed the sensitivity and specificity of each test. The results were also in accordance with the other studies conducted in different parts of India [10-13]. The cefoxitin disc diffusion method has 100% sensitivity and 100% specificity, which is the most suitable method in the detection of MRSA in place of PCR detection of the mecA gene. Clinical Laboratory Standards Institute (CLSI) also has recommended Cefoxitin disc diffusion as the surrogate marker for the detection of MRSA in *S. aureus* strains [5].

The sensitivity and specificity of Oxacillin disc diffusion were 83.7% and 100%, respectively. Although the sensitivity and specificity are high, the detection of heteroresistance isolates of *S. aureus* was difficult. These results are also in accordance with the various studies [14,15].

Mannitol salt agar with 6µg/ml showed sensitivity and specificity as 70.3% and 100%, respectively. A similar study showed sensitivity and specificity as 98.1% and 95.1%, which has a higher sensitivity rate compared to our study [6]. In another study, the Mannitol salt agar with 4µg has showed lesser sensitivity of 66% [16]. The Mannitol salt agar with 6µg also has difficulty in detecting heterogeneous resistant strain due to low expression of resistance. Thus, this test is found to be ancillary when compared to other phenotypic methods.

In our study, it was found that all *S. aureus* were sensitive to both Vancomycin and linezolid, similar to various studies in India and other parts of the world [17].

Among 100 *S. aureus* isolates, 87% were resistant to Penicillin, 40% were resistant to Gentamicin, 27% were resistant to Co-trimaxozole, 32% were resistant to ciprofloxacin [18-20]. In our study, the overall rate of Inducible Clindamycin resistance among *S. aureus* was 24% and 37.2% in MRSA isolates and 15.8% among MSSA isolates [21,22]. This shows that there is co-resistance with the Macrolides, which makes limited treatment options for MRSA infections.

## Conclusions

The increasing trend of MRSA prevalence has urged the clinical laboratory to identify MRSA, which would be rapid and accurate even in resource-limited laboratories. MRSA by mecA gene detection, the “Gold standard” to confirm ambiguous results, is difficult to perform in routine diagnostic laboratories. The present study revealed that among the phenotypic methods, the Cefoxitin disc diffusion method has high sensitivity and specificity compared to other methods for the detection of MRSA. 'D-test' is another additional test to choose the appropriate antibiotic for MRSA infection. 'D-test' is undoubtedly important to characterize inducible Clindamycin resistance from Constitutive Clindamycin resistance. So, a D-test is also suggested along with routine antibiotic susceptibility as it is easy, cost-effective, and reliable. The above methods can be preferred in clinical microbiological laboratories because they are easy to perform, do not require special techniques like specified temperature, special media, and are cost-effective in comparison to other methods.
